# Circulating Angiogenic Markers in Gastroenteropancreatic Neuroendocrine Neoplasms: A Systematic Review

**DOI:** 10.3390/cimb44090274

**Published:** 2022-09-04

**Authors:** Irina Sandra, Irina Mihaela Cazacu, Vlad Mihai Croitoru, Mariana Mihaila, Vlad Herlea, Mircea Mihai Diculescu, Simona Olimpia Dima, Adina Emilia Croitoru

**Affiliations:** 1 Faculty of Medicine, “Carol Davila” University of Medicine and Pharmacy, 020022 Bucharest, Romania; 2 Department of Oncology, Fundeni Clinical Institute, 022328 Bucharest, Romania; 3 Faculty of Medicine, “Titu Maiorescu” University of Medicine and Pharmacy, 020022 Bucharest, Romania; 4 Department of Internal Medicine, Fundeni Clinical Institute, 022328 Bucharest, Romania; 5 Department of Pathology, Fundeni Clinical Institute, 022328 Bucharest, Romania; 6 Department of Gastroenterology and Hepatology, Fundeni Clinical Institute, 022328 Bucharest, Romania; 7 Department of General Surgery, Fundeni Clinical Institute, 022328 Bucharest, Romania; 8 Center of Excellence in Translational Medicine, Fundeni Clinical Institute, 022328 Bucharest, Romania

**Keywords:** neuroendocrine tumors, gastroenteropancreatic, angiogenic, biomarkers

## Abstract

Background: Neuroendocrine neoplasms are a heterogeneous group of tumors that raise challenges in terms of diagnosis, treatment and monitoring. Despite continuous efforts, no biomarker has showed satisfying accuracy in predicting outcome or response to treatment. Methods: We conducted a systematic review to determine relevant circulating biomarkers for angiogenesis in neuroendocrine tumors. We searched three databases (Pubmed, Embase, Web of Science) using the keywords “neuroendocrine” and “biomarkers”, plus specific biomarkers were searched by full and abbreviated name. From a total of 2448 publications, 11 articles met the eligibility criteria. Results: VEGF is the most potent and the most studied angiogenic molecule, but results were highly controversial. Placental growth factor, Angiopoietin 2 and IL-8 were the most consistent markers in predicting poor outcome and aggressive disease behavior. Conclusions: There is no robust evidence so far to sustain the use of angiogenic biomarkers in routine practice, although the results show promising leads.

## 1. Introduction

Neuroendocrine neoplasms (NENs) comprise a group of genetically and clinically diverse epithelial tumors derived from the neuroendocrine system, with primary sites located in most organs [1]. In 50% of cases, primary tumors are located in the gastrointestinal tract and pancreas [2]. Their presence is considered uncommon, even though the age-adjusted incidence has doubled in the last two decades from 2.48 to 5.86 per 100,000/year, mostly due to improved detection and awareness [2,3].

Until recently, NENs were an obscure disorder, due to the scarcity of knowledge and limited therapeutic options. The rapid growth of interest in recent decades led to remarkable progress, with many countries developing networks of multidisciplinary experts dedicated to the management of NENs, starting with diagnosis. 

Apart from its low prevalence, NENs feature intriguing particularities that stand out from other malignancies. Around 40% of these tumors retained their original capacity to synthesize and excrete bioactive molecules, such as insulin, gastrin, glucagon or somatostatin, and are associated with symptoms of variable degrees due to hormone overproduction (Neuroendocrine Tumor of the Gastrointestinal Tract: Introduction, 2021). Another distinctive characteristic is the wide range of behaviors, from completely dormant tumors to aggressive progression and metastasis. It appears that pancreatic neuroendocrine neoplasms (pNENs) exhibit a slightly worse outcome than other gastrointestinal NENs (GEP-NENs), except insulinomas [4]. Furthermore, neuroendocrine tumors benefit from a rich vascularization network compared to other solid tumors [5]. This interesting feature has been proven useful in clinical practice for diagnostic and therapeutic purposes and shows promising perspectives to extend its role as a non-invasive predictive marker. 

Monitoring progression or response to treatment might be challenging, especially in slowly progressive well-differentiated NENs, where radiologic evaluation can be inconclusive. Currently, the most commonly used predictive markers in routine practice are disease stage, tumor cell differentiation and proliferation, quantified by Ki-67 [6]. The last one was included in the three available NEN staging systems (WHO, ENETS and AJCC). However, clinical experience has proven countless times that individuals belonging to the same disease stage evolve differently and respond variably to therapy. One of the major research directions in the field of NENs is identifying non-invasive and reliable biomarkers to circumvent this issue. Initially, efforts were focused on predicting survival and tumor progression (prognostic biomarker). Gradually, studies oriented towards response to treatment [7] and, eventually, to acquired loss of response (predictive biomarker) [8]. 

The aim of this study was to conduct a systematic review of the literature to determine relevant circulant biomarkers of angiogenesis in neuroendocrine tumors.

## 2. Materials and Methods

This systematic review was carried out according to the PRISMA standards. We searched in three databases using the keywords “neuroendocrine” in *title* or *abstract* AND specific biomarkers by full name and abbreviated form in *full text*: vascular endothelial growth factor (VEGF), angiopoietin (Ang), platelet-derived growth factor (PDGFR), basic fibroblast growth factor (bFGF), placental growth factor (PlGF), interleukin (IL)-8 and circulating endothelial cells. 

**Figure 1:**
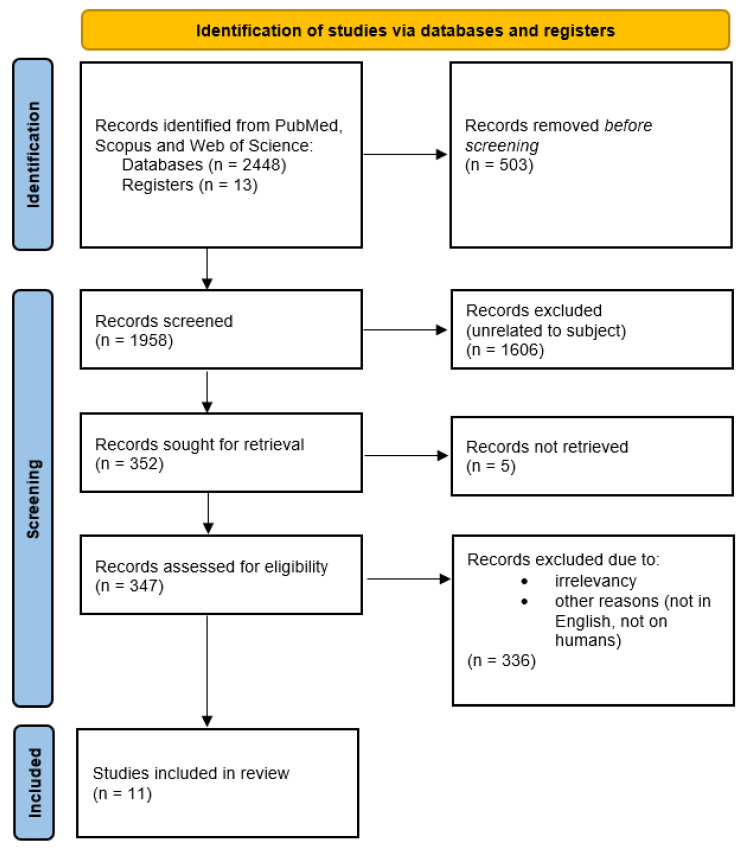
PRISMA diagram for systematic review.

Due to the scarcity of the results, a broader search was conducted using the keywords “neuroendocrine” AND “biomarker” to ensure that all relevant literature was covered. From the search results, only articles including gastroenteropancreatic NEN were selected. The search was last updated on 29 March 2022. In total, 2448 publications were primarily retrieved in PubMed, Scopus and Web of Science. We added 13 after manually searching major scientific journals and from other references. After excluding 503 duplicates, 1958 titles were screened for eligibility and 352 articles were assessed for full-text evaluation. Only human studies were accepted and most articles were excluded due to irrelevancy. We focused mainly on circulating biomarkers; therefore, studies that included only tissular markers or gene expression analysis were excluded. No cut-off point was set for the date of publishing due to the paucity of articles. At the end of the process, 11 articles were included. The search strategy is detailed in Figure 1.

## 3. Results and Discussion

### 3.1. Overview of the Selected Articles

We included 11 articles, most of them originating in Europe. Seven studies were prospective and one was designed with a retrospective and a prospective phase; seven studies included a control group and one was a placebo-controlled clinical trial. Four of the selected studies were clinical trials: two with Sunitinib (phase II and phase IV) [9,10], one with Everolimus (RADIANT 3, phase III) [11] and one with Pazopanib (PAZONET, phase II) [12]. Four studies were published more than 10 years ago. All studies included pNENs and contained previously treated patients. Some studies included extra-digestive tumor sites [12,13,14,15]. Two clinical trials were conducted exclusively on pNENs [9,11]. Biomarkers of interest were: VEGF, angiopoietin, bFGF, PlGF, IL-8, endostatin, stromal cell-derived factor-1 (SDF-1a) and circulating endothelial cells. All studies focused on progression-free survival (PFS), overall survival (OS) and response to treatment as primary or secondary endpoints. Further details on selected studies are summarized in Table 1 and Table 2.

### 3.2. The Current State of Biomarkers in NENs and Future Perspectives

To date, a plethora of markers has been studied and some correlated more or less ideally with tumor outcome. Classic radiological, clinical and pathological features (such as tumor grade, differentiation degree, proliferation status, neurovascular invasion, functionality and tumor spreading) are invaluable parameters to provide a basic overview and select the appropriate strategy in routine practice [6]. Assessment of common serum NEN markers is equally endorsed by guidelines [20], but the results must be interpreted with caution. 

Even though the granin family consists of at least three members (chromogranin A, B and C), all secreted by neuroendocrine cells, only chromogranin A (CgA) has proven its utility in the diagnosis of NENs [21]. Its dynamic also correlates significantly with progression [22], survival [23] and response to treatment [24], making it the most valuable serological biomarker in NENs at the moment. Still, multiple conditions are falsely associated with an up to 10–15-fold increase in CgA, of which the most frequent are hypergastrinemia, renal insufficiency and use of proton-pump inhibitors [25]. Except for serotonin and its metabolite 5-hydroxyindoloacetic acid (5HIAA) in evaluating carcinoid syndrome [26], other serological biomarkers (for example: neuron-specific enolase, pancreatic polypeptide, N-terminal probrain natriuretic peptide) are inferior to CgA as diagnostic or predictive biomarkers [26,27,28]. Incremental progress has been noted in functional imaging as well, culminating in the emerging field of theranostics, which currently refines scoring systems that aim to better predict staging and clinical outcome [29]. Response to somatostatin analogs (SSAs) and peptide receptor radionuclide therapy is classically predicted using radiolabeled SSAs. Moreover, dynamic contrast-enhanced magnetic resonance imaging has shown promising leads in predicting response to anti-angiogenic treatment, even determining optimal biological drug dose [30]. 

Looking forward, we are on the verge of an inflection point where modern techniques, such as genome-wide expression profiling or liquid biopsy, are becoming more accessible. Next-generation sequencing has provided not only insights into the pathophysiology of NENs, but also potential targetable mutations and prognostic markers [31,32]. NETest (Wren Laboratories, Branford, CT, USA) is a polymerase chain reaction (PCR)-based multianalyte algorithmic assay using blood or tissue samples [33] that shows the most promising results so far in terms of predicting diagnosis, outcome and response to treatment [33,34,35], with the potential to detect tumor progression up to 2 years before radiological changes [36]. Circulating tumor cells, free circulating DNA and microRNA testing has been extrapolated in NENs and shown some potential, although further research is required [37,38,39,40]. Despite encouraging evidence, no biomarker has yet ideally met the need to accurately predict tumor behavior. 

### 3.3. Angiogenesis in Neuroendocrine Tumors

Hypervascularization is a hallmark of NENs that has been explored for diagnostic and therapeutic purposes. Neoangiogenesis in NEN is based on several adaptive features that are specific to tumor cells in order to sustain the need of the rapidly developing cells. Notably, while normal tissue relies on the intricate mechanisms of sprouting and intussusception (the division of a preexisting vessel), tumor tissue has developed other pathological models in response to a hypoxic environment, of which the most important are [41]:

− Vessel co-option: preexisting vessels are “hijacked” to serve tumor cells.
− Vasculogenic mimicry: tumor cells build blood channels similar to the endothelium [42].

These mechanisms not only sustain a “comfortable” environment for tumor growth, but they are also responsible, at least in part, for eluding anti-angiogenic therapy [43]. These models are more relevant in high-grade NENs, where hypervascularization is triggered mostly by hypoxia through signaling pathways common with other malignancies [44,45]. 

In well-differentiated NENs, neoangiogenesis appears to be triggered by the autocrine/paracrine secretion of vascular endothelial growth factor (VEGF), regardless of tissular oxygenation. This function is inherited from normal neuroendocrine cells that constitutively synthesize VEGF and release it into the blood flow [42]. This phenomenon has been better described in pNENs as “neuroendocrine paradox”, where low-grade tumors with less aggressive behavior bear the richest vascularization [46]. 

Further unexpected behavior was observed in experimental mouse models showing that liver metastases do not rely on active angiogenesis until advanced stages when a certain tumor volume is reached [46]. This raises a question regarding the propitious timing for anti-angiogenic treatment [42].

### 3.4. The Vascular Endothelial Growth Factor Family

The vascular endothelial growth factor family and their receptors (VEGF-VEGFR/Neurolipins) represent the main proangiogenic molecules. VEGFA (also referred to as VEGF) is the first detected angiogenic molecule and the most potent [47]. It binds to VEGFR-2, resulting in the most efficient signaling to neurolipins that potentiate this angiogenic effect and to VEGFR-1 (or Flt-1) for a weaker activity [43], to the point that a reversed effect was reported by trapping its ligand [48]

Since an overwhelming proportion of angiogenesis is governed by VEGF and its signaling pathways [43], it is natural that most studies address the members of this particular family. Pavel et al. [13] were the first to analyze circulating angiogenic markers in NENs. The group consisted of 38 patients with digestive and extra-digestive primary site, mostly low grade and metastatic. VEGF levels were significantly higher in NENs compared to healthy controls, irrespective of site, grade or functionality status. On a short-term period, the dynamics of VEGF were as reliable as CgA (*p* < 0.0001) in predicting disease progression (*p* < 0.011), even though fewer samples were available for serial analysis. It is reasonable to infer that results were hardly impacted by treatment, as most pre-treated patients were on octreotide and mean VEGF levels were similar between treated and naïve patients. However, the negative influence of SSAs on VEGF levels was reported [49,50]. VEGF serum levels did not correlate with OS. 

Worse outcome in patients with high VEGF levels was not confirmed in a larger study by Berkovic et al. [16]. Higher median levels of VEGF were reported in GEP-NENs, but inversely correlated with histologic differentiation and aggressiveness (notably with Ki-67 rather than grade). This is suggestive of the “neuroendocrine paradox” described in pNENs [42] but OS was not assessed in the study. Interestingly, while circulating VEGF levels were increased in metastatic disease, the highest levels were noted in lymph node metastases and the lowest in liver metastases. Berkovic et al. broke new ground with a multi-directional approach in studying the role of VEGF in GEP-NENs. They were the first to study the role of VEGF 1154A/G polymorphism in GEP-NENs. Higher VEGF serum levels were associated with the -1154G allele, thus, inferring a potential involvement of VEGF-1154 SNP (single nucleotide polymorphism) in VEGF expression, in line with the ones generated by other cancers [51,52]. However, further research is required, as the correlation was statistically significant only in non-functional non-pancreatic gastrointestinal NENs. 

Five more studies included VEGF (Grande et al., Yao et al., Zurita et al., Melen-Muncha et al., Figueroa-Vega et al.), but no statistical significance was demonstrated concerning the study objectives, although two of the clinical trials involved large sample sizes.

Three clinical trials analyzed VEGFR (Zurita et al., Yao et al., Grande et al.). Zurita et al. [10] were the first to report predictive value for better survival in pancreatic (and not intestinal) well-differentiated NEN patients with high levels of VEGFR-2 at baseline (*p* = 0.01). No significant correlation with PFS was found. In carcinoid tumors, VEGFR-3 predicted poor outcome in terms of PFS (*p* = 0.006) and OS (*p* = 0.047). Yao et al. and Grande et al. did not report clinical correlations with VEGF receptors. Jiménez-Fonseca et al. [9] additionally tested 14 SNP variants in nine genes, including VEGFR-3. None of them were associated with outcome after applying multivariate analysis. Three other VEGFR-3 SNP variants correlated with shorter OS (rs307826 and rs307821, Zurita et al.) and shorter PFS (rs307821, Grande et al.). 

### 3.5. Placental Growth Factor

Placental growth factor (PlGF) has a similar structure to VEGF, but it becomes active only in pathological conditions [53]. It appears to bind selectively to VEGFR-1 and neurolipins, but not to VEGFR-2, thus, increasing the availability of VEGF for its most efficient pathway [54]. Hilfenhaus et al. [19] conducted the most comprehensive study on PlGF regarding the function, the prognostic value and the potential of a single marker as a therapeutic target, with encouraging results. The study was designed to benefit from a multi-level approach: clinical analysis in serum and tissue samples collected retrospectively and prospectively, in vitro analysis using a panel of cell lines confirmed with PlGF receptors and in vivo analysis on mouse models. The study showed compelling clinical and preclinical evidence for the pivotal role of PlGF in the tumorigenesis of well-differentiated NENs. Higher levels were reported in NENs and correlated unexpectedly with grading, but not with metastatic disease. Further analysis confirmed the role of PlGF as a prognostic marker of poor outcome (indicative of shorter survival in pNENs and shorter time to progression in intestinal NENs). 

An in vitro experiment not only confirmed the role of PlGF in tumor aggressiveness by stimulating proliferation and cell migration but also hypothesized that PlGF might be useful in monitoring the short-term response to angiogenic treatment (levels raised significantly in response to Sunitinib). Finally, in vivo experiment on mouse models inoculated with pNENs xenografts showed important reduction in tumor volume after PlGF inhibition, thus, revealing the opportunity of a new therapeutic target. 

In a classical trial RADIANT 3, a panel of markers was assessed in a large group of well-differentiated pNENs treated with Everolimus (PlGF, VEGF-A, VEGFR1, VEGFR2, bFGF). After applying multivariate analysis, only PlGF emerged as an independent prognostic factor for disease progression. 

### 3.6. Angiopoietin-Tie-2

Angiopoietin (Ang)-Tie: Ang-1 acts as an agonist to Tie-2, promoting vascular stability. Ang-2 is expressed in pathological conditions in response to proangiogenic molecules and functions as a context-depending antagonist, competing for the same receptor Tie-2 [55]. 

Srirajaskanthan et al. [14] focused on analyzing two of the most relevant exponents of the angiopoietin family (Ang-1 and Ang-2) in a group of well-differentiated NENs having different primary sites. They were the first to demonstrate higher levels of Ang-2 in NENs compared to healthy controls (*p* < 0.001), proportional to tumor burden (*p* = 0.014, one-way ANOVA test), with a sensitivity close to that of CgA. Despite short follow-up (6 months), Kaplan–Meier curves showed a statistically significant correlation with a shorter time to disease progression (*p* = 0.03). No particular pattern was noticed in Ang-1 levels, with the caveat that the group was highly heterogenous in terms of tumor site, previous treatment and tumor extension. Ang-2 was also assessed in nine tissue samples; however, the small sample size and intra-tumoral variation in staining hampered the conclusions. 

Detjen et al. [17] extended research on clinical and physiological implications of Ang-2 in NENs in a holistic manner, implying circulant levels, tissue immunohistochemical staining and in situ hybridization and preclinical mouse model using orthotopic xenografts. Clinical findings were of particular interest. Prognostic potential for unfavorable outcome in high-serum Ang-2 group (confirmed also in the previous study) was extended to survival (median of 13 months versus undefined in low/medium Ang-2, *p* = 0.0003). It appears that Ang-2 is more reliable in predicting poor survival than the presence of metastases. Furthermore, expression patterns of Ang-2 mRNA and receptor Tie-2 in a small subset of tissue samples provided insights into the physiology of the Ang-2–Tie-2 axis in NENs, in favor of a de novo autocrine or paracrine secretion of Ang-2. Finally, the experimental mouse model highlighted the stimulating effect of Ang-2 on angiogenesis and metastasis in the subgroup of pNENs. On the contrary, Ang-1 levels did not correlate with Ang-2, nor did they provide significant clinical correlations. 

Controversial data are brought by Figueroa-Vega et al. [18], regarding Ang-1, Ang-2 and Tie-2 expression in blood and tissue samples. Higher levels of all three analyzed markers were reported in metastatic GEP-NENs when compared to healthy controls; however, neither was effective in predicting the risk of metastasis, vascular invasion and response to SSAs. Histological expression of these markers equally failed to demonstrate significant relevance, most probably due to the small sample size. The original aspect of this study design is the assessment of Tie-2-expressing monocytes in well-differentiated GEP-NENs, which brings valuable knowledge on the physiology and potential therapeutic relevance of the Ang-Tie-2 axis. Tie-2-expressing monocytes were significantly increased in tumor cells (*p* < 0.05), with enhanced chemotaxis, demonstrated in vitro in response to Ang-2 but not to Ang-1, nor in the presence of blocking antiTie-2 antibodies. 

Melen-Mucha et al. [15] assessed a larger panel of markers in plasma from 36 well-differentiated GEP NENs patients. The particular choice of using plasma samples is not arbitrary, as platelets are known to artifact the results. Very few markers showed statistically significant relevance. Only Tie-2 levels were elevated in NENs compared with healthy controls (*p* < 0.001), proving to be as effective as CgA as diagnosis markers. Tie-2 did not correlate with the outcome of the disease. Elevated levels of Ang-2 were once again confirmed in metastatic disease (*p* < 0.05). 

### 3.7. Other Molecules

IL-8 is a pro-inflammatory chemokine that exerts a strong angiogenic effect as a chemotactic and growth factor for endothelial cells [56]. It has been studied in numerous cancers [57,58,59], including adenocarcinomas of the pancreas [60] but data on NENs are scarce. Pavel et al. [13] demonstrated that low baseline levels of IL-8 correlated not only with disease progression (*p* < 0.008), but also with poor survival (*p* = 0.009) [13]. Notably, none of the non-survivors at 2 years had baseline levels below the detection limit. One particular strength of the study conducted by Pavel et al. is leveraging the use of ultra-sensitive ELISA to observe higher levels of IL-8 also in stable disease (*p* < 0.037), which was not obvious otherwise. Moreover, in two clinical trials, low baseline IL-8 predicted a better response to Sunitinib in carcinoids (Jiménez-Fonseca et al.) and pNENs (Zurita et al.). High baseline levels were also prognostic for shorter PFS and OS (Zurita et al.). Moreover, Jiménez-Fonseca et al. observed, during follow-up, increasing levels of IL-8 in patients resistant to Sunitinib, suggesting that it may be a part of the mechanism of resistance to treatment.

Stromal-cell-derived factor 1a (SDF1a) is a ubiquitous cytokine that recruits endothelial progenitor cells during angiogenesis [61]. Zurita et al. assessed SDF-1a in a subgroup of 28 patients and correlated high levels with poor outcome in terms of progression and survival. As such, 22 out of 28 died during follow-up.

Zurita et al. also demonstrated that CD14^+^ monocyte co-expressing VEGFR-1 or CXCR4 significantly lowered during treatment with Sunitinib (*p* = 0.04 and *p* = 0.03, respectively), suggesting that they serve as predictors of response. 

bFGF was analyzed in two studies (Pavel et al., Yao et al.) but the results failed to show any clinical correlations. 

In Pavel et al.’s study, angiogenin failed to demonstrate any prognostic value, although angiogenin was significantly higher in NENs compared to controls (*p* < 0.003) [13].

In spite of sustained endeavor toward research, there is no compelling evidence to sustain the use of angiogenic markers in clinical practice for prognostic or predictive purposes. The majority of studies face several limitations that impact the reliability of the results. It is worth reiterating that a small sample size is the major issue in studying such rare diseases. Except for two clinical trials, only one study in our selection exceeded 100 patients. At the same time, the population is usually heterogenous in terms of primary site, staging and grading, and the majority of patients have already benefited from at least one treatment. 

Another issue regards the lack of data on G3 NENs for two main reasons. Most studies focused on well-differentiated tumors G1 or G2 for understandable reasons (only two studies included G3). Secondly, since all studies were conducted before the most recent WHO classification (2017), well-differentiated G3 tumors were not part of the picture.

## 4. Conclusions

Although data are not mature yet, there are still some promising leads in the field of angiogenic markers. Statistically significant correlations with tumor behavior or outcome were reported for markers, such as PlGF, Ang-2 or IL-8, in at least two independent studies, suggesting attractive leads for deeper research. In theory, all therapeutically targetable molecules additionally motivate further effort. For future perspectives, it is possible that a meta-analysis would overcome the limitations of size and heterogeneity. Moreover, since single predictors are insufficient, the next step in the research should include multianalyte biomarkers.

## Figures and Tables

**Table 1:** Study design.

Author, Journal, Year	Country	Study Type	Enrollment Period	Follow-Up (Months)	Control Group	Sample size	Endpoints	Biomarkers	Source	Technique (for Circulating Markers)
Pavel et al., Clin Endocrinol, 2005 [13]	Germany	retrospective	June 1999–July 2002	24	yes	38	Progression and OS	VEGF, IL-8, bFGF, Angiogenin	blood	ELISA, usELISA
Berković et al., Mol Cell Endocrinol, 2016 [16]	Croatia	prospective	NA	NA	yes	145	Correlation with DP; The role of VEGF 1154 SNPs in VEGF expression	VEGF, VEGF 1154A/G polymorphism	blood tissue	ELISA
Srirajaskanthan et al., Endoc Rel Cancer, 2009 [14]	UK	prospective	July 2007–March 2008	6	yes	47	Correlation with DP and PFS	Ang-1, Ang-2	blood tissue	ELISA
Derjen et al., Clin Cancer Res, 2010 [17]	Germany	prospective	1998–2005	59	yes	42	Correlation with DP and OS; Physiological implications of Ang-2	Ang-2	blood tissue xenograft	ELISA
N Figueroa- Vega et al., Endocr Rel Cancer, 2010 [18]	Spain	retrospective	NA	NA	yes	47	Correlation with DP and response to treatment. Physiological implications of Ang 1 and 2 -Tie 2 axis	Tie-2, VEGF Ang-1, Ang-2, TEM	blood tissue	ELISAFACS
Melen-Mucha et al., Int. J. Mol. Sci, 2012 [15]	Poland	retrospective	May 2008–February 2011	NA	yes	36	Correlation with DP	VEGF, Ang-1, Ang-2, Tie-2, Endostatin, osteopontin	blood	ELISA
Hilfenhaus et al., Endocr Rel Cancer, 2013 [19]	Germany	retrospective & prospective	Retrospective: 1998–2012 Prospective: May 2009 December 2012	NA	yes	175	Expression, function, prognostic value and potential therapeutic target	PlGF	blood tissue in vitro xenograft	ELISA
Jiménez-Fonseca et al., Oncotarget, 2018 [9]	Spain	prospectivemulticenterphase IV clinical trial with Sunitinib	November 2012— February 2015	51	No	43	Correlation with OS, PFS, response to treatment, adverse events	panel of 14 SNPs, HGF, IL-6, IL-8, TIMP1, sE-selectin, osteopontin	blood	multiplex bead assays
Zurita et al., BJC, 2015 [10]	USA	prospectivemulticenterphase II clinical trial with Sunitinib	March 2003–November 2005	NA	No	105	Correlation with OS, PFS, response to treatment, adverse events	VEGF-A, VEGFR-2, VEGFR-3, IL-8, SDF-1α, Circulating myelomonocytic and endothelial cells	blood	ELISA FACS
Yao et al., J of Clin Oncol, 2016 “RADIANT-3” [11] (extension phase)	Multi- based	prospective, randomized, placebo-controlled, phase III, initially double-blind then open-label clinical trial with Everolimus	July 2007– March 2014	NA	Initially double blind	Placebo: 203 Everolimus: 207	Correlation with OS	PlGF, VEGF-A, VEGFR1, VEGFR2, bFGF	blood	ELISA
Grande et al., Annals of Oncology, 2015 [12] “PAZONET”	Spain	prospectiveopen-label, phase II clinical trial with Pazopanib	January 2011– March 2012	17	No	44	Correlation with OS, PFS, response to treatment, adverse events	VEGF-A, VEGFR-2, CTCs, CECs, cytochrome P450 3A5, VEGFR3 SNPs	blood tissue	ELISA Cell Search

NA = non-available information; PFS = progression free survival; OS = overall survival; DP = disease phenotype; FACS = flow cytometry analysis; ELISA = enzyme-linked immunosorbent assay; usELISA = ultrasensitive ELISA; VEGF = vascular endothelial growth factor; VEGFR = vascular endothelial growth factor receptor; Ang = angiopoietin; TEM = TIE-2 expressing monocytes; PlGF = placental growth factor; HGF = hepatocyte growth factor; TIMP1 = tissue inhibitor of metalloproteinase-1; bFGF = basic fibroblast growth factor; CTSs = circulant tumor cells; CECs = circulant endothelial cells; SNPs = Single nucleotide polymorphisms.

**Table 2:** Study characteristics.

Author	Primary site	Metastatic	Previous treatment	Conclusions
Pavel et al., Clin Endocrinol, 2005 [13]	pNENs: 11 GI-NENs: 13 Other: 14	37 (97%)	pretreated: 22 treatment naïve: 16	↑ VEGF levels correlated with PD ↓ baseline IL-8 correlated with PD and ↓ OS
Berković et al., Mol Cell Endocrinol, 2016 [16]	pNENs: 65 GI-NENs: 80	58 (40%)	NA	↑ VEGF in GEP-NENs, particularly in lymph node metastases and secretory status.
Srirajaskanthan, et al., Endoc Rel Cancer, 2009 [14]	pNENs: 17 GI-NENs: 22 Other: 8	NA	pretreated: 43 treatment naïve: 4	↑ Ang-2 in NETs proportional to tumor burden and prognostic of poorer outcome
Detjen et al., Clin Cancer Res, 2010 [17]	pNENs: 25 GI-NENs: 15 Unknown: 2	28 (66%)	NA	↑ Ang-2 in metastatic NETs and prognostic for ↓ OS
Figueroa-Vega et al., Endocr Rel Cancer, 2010 [18]	pNENs: 23 GI-NENs: 12 Other: 12	28	NA	↑ Ang-1, Ang-2 and Tie-2 in GEP NENs, without prognostic relevance; Ang-2 stimulates TEM recruitment at tumor site
Melen-Mucha et al., Int. J. Mol. Sci, 2012 [15]	pNENs: 2 GI-NENs: 12 Other: 22	27 (75%)	pretreated: 14 (with SSA) treatment naïve: 22	↑Tie-2 in NET↑ Ang-2 in metastatic disease
Hilfenhaus et al., Endocr Rel Cancer, 2013 [19]	pNENs: 118 GI-NENs: 57	155	pretreated: 85 naïve: 90	↑ PlGF in NET. Correlation with grading, not metastases. ↑ VEGFR1 in metastatic disease. ↑ PlGF predicted ↓ OS in pNETs (not confirmed in multivariate analysis) and shorter time to progression in GI-NETs. In vivo: significant reduction in tumor volume after PlGF inhibition
Jiménez-Fonseca et al., Oncotarget, 2018 [9]	All pNEN	42 (97%)	pretreated: 25 treatment naïve: 18	2 VEGFR-3 SNP (rs307826 and rs307821) associated with ↓ OS↓ IL-8 associated to better objective response
Zurita et al., BJC, 2015 [10]	pNENs: 66 Carcinoid: 39	NA	NA	↑ VEGFR-2 predicted ↑ OS in pNETs↓ IL8 predicted response to treatment in carcinoid↑ VEGFR-3 and IL-8 correlated with ↓ PFS and ↓ OS in carcinoid↑ SDF-1α predicted ↑ PFS and ↓ OS
Yao et al., J of Clin Oncol, 2016 [11] “RADIANT-3” (extension phase)	All pNEN	NA	NA	PlGF is an independent prognostic factor for PD
Grande et al., Annals of Oncology, 2015 [12] “PAZONET”	pNENs: 18 GI-NENs: 15 Other: 11	NA	all	No significant correlation with circulant biomarkers was noted; VEGFR3rs307821 correlated with ↓ PFS in GEP NET

pNENs = pancreatic neuroendocrine neoplasms; GI-NENs = gastro-intestinal neuroendocrine; GEP-NENs = gastro-entero-pancreatic neoplasia; NETs = (well differentiatied) neuroendocrine tumors; ↑ = increased; ↓ = decreased; PD = progressive disease; OS = overall survival; NA = information not available; VEGF = vascular endothelial growth factor; VEGFR = vascular endothelial growth factor receptor; Ang = angiopoietin; TEM = TIE-2 expressing monocytes; SNPs = Single nucleotide polymorphisms; PlGF = placental growth factor; SSA = somatostatin analogues.

## Data Availability

Not applicable.
